# From Corrective to Predictive Maintenance—A Review of Maintenance Approaches for the Power Industry

**DOI:** 10.3390/s23135970

**Published:** 2023-06-27

**Authors:** Marek Molęda, Bożena Małysiak-Mrozek, Weiping Ding, Vaidy Sunderam, Dariusz Mrozek

**Affiliations:** 1 TAURON Wytwarzanie S.A., Promienna 51, 43-603 Jaworzno, Poland; marek.moleda@gmail.com; 2 Department of Distributed Systems and Informatic Devices, Silesian University of Technology, 44-100 Gliwice, Poland; bozena.malysiak@polsl.pl; 3 School of Information Science and Technology, Nantong University, No. 9 Seyuan Road, Nantong 226019, China; dwp9988@163.com; 4 Department of Computer Science, Emory University, Atlanta, GA 30322, USA; vss@emory.edu; 5 Department of Applied Informatics, Silesian University of Technology, 44-100 Gliwice, Poland

**Keywords:** power industry, energy production, predictive maintenance (PdM), prognostics and health management (PHM), smart maintenance, Industry 4.0

## Abstract

Appropriate maintenance of industrial equipment keeps production systems in good health and ensures the stability of production processes. In specific production sectors, such as the electrical power industry, equipment failures are rare but may lead to high costs and substantial economic losses not only for the power plant but for consumers and the larger society. Therefore, the power production industry relies on a variety of approaches to maintenance tasks, ranging from traditional solutions and engineering know-how to smart, AI-based analytics to avoid potential downtimes. This review shows the evolution of maintenance approaches to support maintenance planning, equipment monitoring and supervision. We present older techniques traditionally used in maintenance tasks and those that rely on IT analytics to automate tasks and perform the inference process for failure detection. We analyze prognostics and health-management techniques in detail, including their requirements, advantages and limitations. The review focuses on the power-generation sector. However, some of the issues addressed are common to other industries. The article also presents concepts and solutions that utilize emerging technologies related to Industry 4.0, touching on prescriptive analysis, Big Data and the Internet of Things. The primary motivation and purpose of the article are to present the existing practices and classic methods used by engineers, as well as modern approaches drawing from Artificial Intelligence and the concept of Industry 4.0. The summary of existing practices and the state of the art in the area of predictive maintenance provides two benefits. On the one hand, it leads to improving processes by matching existing tools and methods. On the other hand, it shows researchers potential directions for further analysis and new developments.

## 1. Introduction

Maintenance is an indispensable part of almost every production process in industry. The knowledge base and current procedures in this area have evolved since the first industrial revolution. Thus, how can modern technology support current maintenance practices and what benefits does it provide? According to a Delloite report, inappropriate maintenance strategies can reduce overall production capacity by 5 to 20 percent [1]. Likewise, McKinsey forecasts that digital maintenance in the industry can increase asset availability by 5 to 15 percent and reduce maintenance costs by 18 to 25 percent [2]. The top use cases of modern technologies in the industry relate to maintenance. These are predictive maintenance and inspection quality [3]. The main value drivers for using these approaches are as follows [4]:

- Uptime improvement;
- Cost reduction;
- Improved safety, health, environment and quality;
- Extension of asset life.

Digitization and transformation of predictive processes allow a better understanding of the occurring processes and enable more accurate and justified decisions that rely less on intuition. Furthermore, knowledge from integrated data sources and advanced analytics enables implementing new maintenance strategies, better work and inventory planning, increased production efficiency and increased safety levels.

The power industry has undergone important transformations in recent years, driven by new energy sources, climate changes and ecological factors. This sector can therefore benefit from modern approaches to keep equipment working properly and avoid contamination of the environment and to prevent failures that may have serious ecological consequences. This review presents maintenance techniques and strategies that can help prevent failures and reduce the risk of such problems in the power industry.

In this article, we consider the issue of digital transformation in the maintenance arena with a particular focus on the energy industry. The review will explore how we can improve maintenance by considering opportunities offered by Industry 4.0. The scope includes applications in the energy industry or those that can be adapted from other industry sectors, both in the area of centralized units such as power plants and distributed renewable energy installations.

### 1.1. Energy Industry

Power engineering is the branch of the industry dealing with the production and distribution of electric and thermal energy. Based on the generation method, we can divide the production of energy into conventional (thermal, gas power plants) and unconventional (wind farms, photovoltaic panels, hydroelectric power plants). Generation methods are strongly linked to energy sources, which can be renewable (water, wind, solar, biomass) and non-renewable (natural gas, coal). The structure of generating assets should ensure high availability and reliability since electricity cannot be stored, i.e., the current temporary production should correspond to the actual demand of recipients. A particular challenge is to increase the share of renewables due to environmental requirements, which necessitates new maintenance techniques for these facilities and unique operating characteristics.

Depending on the type of plant, the maintenance process has to face different typical faults and different operation specifics. In the case of a thermal power plant, most of the equipment is concentrated in a small area. Then, the maintenance process is particularly directed at maintaining the equipment involved in production, such as boilers, feed pumps, turbines and generators. Typical faults can be primarily divided into:

- Mechanical faults: bearing damage, vibration, heating;
- Automation system faults: control system malfunctions, tripping of safety systems;
- Water and oil leaks from machinery and pipes;
- Fuel supply failures: mills, conveyor belts.

Renewable energy sources and distribution networks are characterized by dispersed distribution, which entails the need for rational planning of maintenance work and monitoring asset health. The cost of inspection and maintenance work in the case of, for example, a wind turbine is associated with higher costs related to safety, logistics and necessary capacities.

**Figure 1:**
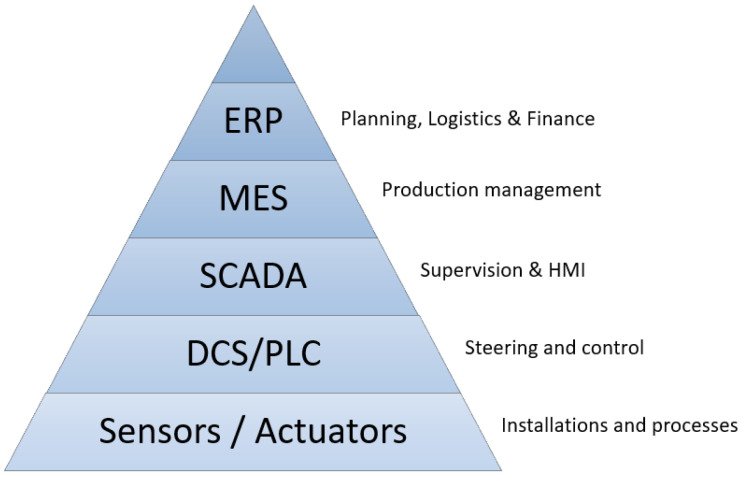
Pyramid of system levels.

Data resources that can be used for maintenance analysis include groups of systems ranging from enterprise to automation control systems. Considering business levels, we can distinguish the following types of layers in the functioning systems (Figure 1):

- ERP (Enterprise Resource Planning): A group of systems operating at the corporate level, including the systems supporting the implementation of planning, financial and procurement processes of the company.
- MES (Manufacturing Execution System): Systems in this class operate mainly on data from Operational Technology (OT) systems (SCADA, DCS) and are used to monitor and optimize the production process.
- SCADA (Supervisory Control And Data Acquisition): Systems designed to facilitate operator monitoring and control of the production process in real time. This is also a type of HMI (human–machine interface) that allows the operator to interact with the device.
- DCS/PLC (distributed control system/programmable logical controller): Devices controlling the production process in a network connecting sensors, actuators and human–machine interface. They automatically output control signals to devices using data from lower levels.
- Sensors/Actuators: The lowest layer responsible for executing the manufacturing process. It gathers data from sensors, manipulates control signals in real-time networks.

The collection of all data includes both human-created records and machine-generated repositories and logs, which raises some challenges for the data analysis (e.g., *variety* and *volume* that underlie Big Data challenges; see Section 5.2). However, multi-level integration of different systems also opens up new possibilities for insights, transforming existing data assets into real value.

### 1.2. Related Reviews

Although several review papers cover the industrial applications of Industry 4.0 concepts such as predictive maintenance, Big Data and simulation, none covers the traditionally used and modern techniques for maintenance in the energy sector in such a comprehensive manner. A summary and comparison of our review with other papers published in related areas are presented in Table 1. We assumed the following criteria for the comparison:

- Strategy: whether it shows the impact of the presented content on management processes, e.g., strategy formulation or potential change of current processes.
- Methods: whether it presents a detailed description and categorization of methods and algorithms, considering their application, data sources, advantages and disadvantages.
- Diagnosis: whether it includes a description of methods and applications in areas such as fault detection and identification, pattern recognition and root cause analysis.
- Prediction: whether it covers methods and applications in areas such as predictive health management, remaining useful life.
- Prescription: whether it describes advanced analysis applications in the prescriptive area, including techniques such as simulation, digital twin, process optimization.
- Area: whether it presents industries covered by the review.

The papers compared in Table 1 frequently address various issues of Industry 4.0 and predictive maintenance. For example, Carvalho et al. [5], Diez-Olivan et al. [6] and Zonta et al. [7] focus mainly on predictive maintenance (PdM) applications in a wide range of industries. In contrast, Sikorska et al. [8] analyze the methods of remaining useful life, categorizing the features of the approaches in the context of resources and customer requirements. Gao et al. [9,10] focus on presenting FDI methods (fault detection and identification) and describing them in detail, classifying them as model-based and knowledge-based (data-driven). In the area of diagnostics, a comprehensive review of the papers focusing on root cause analysis is presented by Sole [11]. Only a few papers focus on the energy generation field, but even those are specific to particular sources of energy. For example, Fausing et al. [12] gives an overview of predictive maintenance applications in thermal power plants, while Chao et al. [13] and Ngarayana et al. [14] focus on nuclear power plants.

**Table 1:** Related articles with a focus on smart maintenance in the industry (including power-generation industry).

Reference	Year	Strategy	Methods	Diagnosis	Prediction	Prescription	Area	Contribution
Sikorska et al. [8]	2011	○	●	●	●	○	Industry	A comprehensive review of methods related to RUL. Classification of algorithms and presentation of their strengths and weaknesses to facilitate the selection of the suitable model for the specific business required.
Gao et al. [9,10]	2015	○	●	●	○	○	Industry	Survey of fault-diagnosis and fault-tolerance techniques. Classification of methods as model-based, signal-based and knowledge-based (data-driven).
Sole et al. [11]	2017	◑	●	●	○	○	Industry	An overview focused on the root cause analysis problem, taking particular account of requirements, performance and scalability aspects.
Diez-Olivan et al. [6]	2019	○	○	●	●	●	Industry	A review of applications of data-driven predictive algorithms in the industry within the I4.0 paradigm (categorization into descriptive, predictive and prescriptive analysis).
Carvalho et al. [5]	2019	◑	●	◑	◑	○	Industry	A review of ML methods applied to predictive maintenance. Focuses on methods, devices and data sources used.
Zhang et al. [15]	2019	○	●	◑	◑	○	Industry	Focuses on data-driven PdM methods and their applications.
Saufi et al. [16]	2019	○	●	●	○	○	Rotating machinery	A review of deep learning-based methods for fault detection and diagnosis.
Merkt [17]	2019	●	◑	●	●	○	Industry	A review of data-driven predictive methods highlighting challenges and benefits with indicated areas of possible applications.
Alcacer and Cruz-Machado [18]	2019	◑	○	○	○	●	Manufact-uring	Overview of I4.0 technology applications in terms of enabling opportunities and use in manufacturing environments.
Ngarayana et al. [14]	2019	●	◑	◑	◑	◑	Nuclear Power Plant	A review of models, methods and strategies for optimizing maintenance at a nuclear power plant. A comparison of scientific studies with real applications.
Soualhi et al. [19]	2019	○	●	●	○	○	Industry	An overview of diagnostic methods used for fault isolation and identification. Classification of methods as model-based, data-driven and hybrid.
Cinar et al. [20]	2020	○	●	◑	◑	○	Industry	An overview of ML applications in PdM. Classifies papers based on methods, data sources, devices used in data acquisition, data size and critical findings.
Chao et al. [13]	2020	●	○	◑	◑	◑	Nuclear Power Plant	An overview of AI applications categorized for typical scenarios in a nuclear power plant; addresses the problem of human–machine interaction.
Fausing et al. [12]	2020	◑	●	●	●	○	Thermal Power Plant	A review of PdM articles with a focus on the pumping system in power plants.
Zonta et al. [7]	2020	○	◑	●	●	◑	Industry	A systematic literature review of PdM in the industry. Categorizes methods, standards and applications. Discusses the limitations and challenges of PdM.
this article	2022	●	●	●	●	◑	Energy Industry	An overview of data-driven and experience-based methods improving maintenance. Shows applications of advanced analytics in the energy sector.
○: not studied ◑: mentioned ●: studied								

### 1.3. Contributions

Regarding articles dealing with the presented field, we can distinguish between articles focusing on a broad description of methods in the context of the industry as a whole or the industry-specific articles presenting a narrow range of proposed solutions. Issues linking the value of innovative solutions and possible changes to existing procedures, especially strategies, are hardly ever addressed. This paper extends the spectrum of the published works.

- It presents traditional approaches and methods used in maintenance against solutions that extend analytical capabilities and automate handbook processes.
- It shows a wide range of methods covering the areas of diagnostics, prediction and prescription in the context of applications narrowed to the power industry.
- It proposes a simplified classification common to the areas of diagnosis (fault detection and identification) and prognosis (remaining useful life), categorizing groups of methods in two dimensions: model-based/data-driven and qualitative/quantitative.
- It discusses and summarizes the challenges and barriers that limit the use of theoretically proven mechanisms in a production environment in practice.

**Figure 2:**
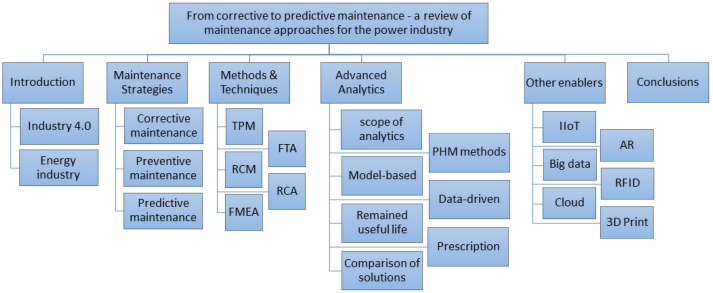
Structure of this review.

The paper is organized according to the outline shown in Figure 2. Section 4 provides an overview of the advanced analytics methods used to support the maintenance area. Here, we describe in detail the algorithms related to fault detection and health index determination. We also present examples of implementations of more complex systems covering prescriptive maintenance and digital twin issues. We describe in detail methods from the area of fault detection and identification, along with the advantages and disadvantages of applications in particular situations. The use cases include detecting specific faults and anomalies, diagnosis to identify problem root causes, prediction of useful life, simulation and optimization of activities. Section 5 describes the possibilities of the remaining concepts of Industry 4.0 concerning the possible applications in the power industry.

## 2. Maintenance Strategies in Industry

Maintaining equipment in good condition is an important issue in the production process. Appropriate service and maintenance contribute to a high level of availability and reduce production downtimes. On the other hand, in power plants, maintenance costs represent a significant financial expense. Therefore, it is essential to achieve acceptable production results while optimizing service costs. According to the definition in the European Standard EN13306 [21], maintenance is defined as a combination of all technical, administrative and managerial actions during the life cycle of an item intended to retain it in, or restore it to, a state in which it can perform the required function. Thus, maintenance includes all activities related to inspections, condition monitoring, routine maintenance, replacement of parts, repairs, overhauls, as well as planning and supervision of all these activities.

**Figure 3:**
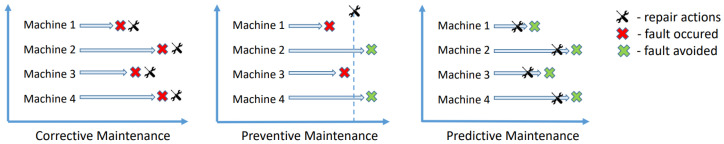
Maintenance strategies showing various moments of the repairing process before and after the potential failure of the equipment (based on [22]).

Maintenance strategies can be classified in terms of the time when a repair is performed relative to the occurrence of a failure. There are three basic approaches of maintenance as shown in Figure 3:

- Corrective;
- Preventive;
- Predictive.

### 2.1. Corrective Maintenance

Corrective maintenance implies taking action after a failure has occurred. This approach minimizes the cost of servicing the equipment, thus extending the maintenance interval, but it comes at the expense of increased risk of equipment unavailability. The negative effects of corrective maintenance may manifest in:

- Lost revenue, increased cost of repairing the equipment or related equipment being more damaged, which is a result of a primary failure;
- Increased time and cost of repair—a result of unplanned downtime.

A simple real-life example of corrective maintenance is replacing a light bulb in a car. The item is only replaced when it burns out, for which the drivers are prepared by having a set of bulbs in reserve.

As stated above, this approach should be used for non-critical, easily repairable equipment. However, a more proactive approach is expected for components whose failure can cause downtime, e.g., steam boiler or turbine in a power plant. The same applies to equipment whose failure may contribute to the degradation or destruction of associated equipment, e.g., conveyor belts in explosion hazardous areas or evaporators or high-pressure steam pipelines. Depending on the procedures defining the moment when the repair should occur, actions can be taken immediately or deferred, depending on the priority and the potential consequences of the failure. In the case of continuous production, a prevalent situation is when some parts permanently work in a defective state. This is difficult to observe since the work parameters of an element slowly deteriorate (which is reflected in, e.g., reduced efficiency, increased vibration, heating). At the same time, they have no or low impact on the efficiency of production.

To facilitate corrective maintenance, we can employ descriptive analytics. Descriptive analytics includes techniques and methods for correctly estimating the failure effects and adopting a proper approach to calculate all potential benefits and risks. When covering equipment with this strategy, it is essential to use diagnostic technologies allowing for fault-detection and -monitoring equipment health. Adopting the proper approach allows taking actions to remove the failure before downtime much quicker.

### 2.2. Preventive Maintenance

The purpose of preventive maintenance is to avoid unplanned downtime through scheduled periodic inspections and replacements. Typically scheduled tasks include lubrication, adjustments, oil changes or advanced diagnostics. Maintenance intervals can be planned on the basis of manufacturer recommendations, analysis of quality parameters such as MTBF (mean time between failure) and MTTF (mean time to failure). Preventive maintenance ensures good equipment condition and reduces the risk of potential downtime. However, it does not protect against unexpected failures and defects of elements not covered by the maintenance. Additionally, replacing parts too often is not always a good option, for two reasons [23]:

- By changing an original part with a replacement, the useful life of the whole unit (machine) could be shortened due to an additional risk of failure of the part, assembly error, hidden defects or non-matching part;
- New parts and consumables have a higher probability of being defective or failing than existing materials that are already in use.

**Figure 4:**
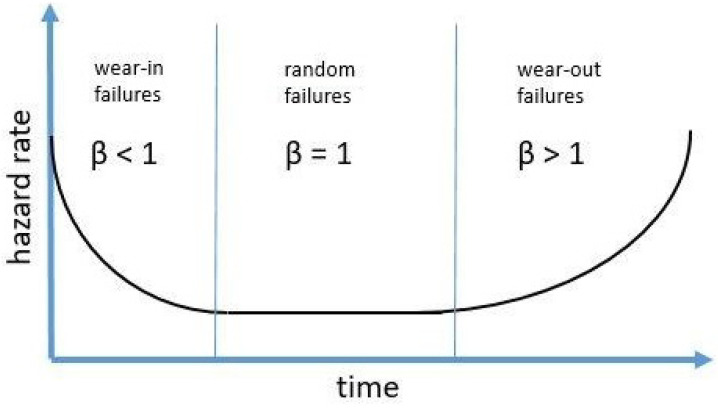
“Bathtub” visualizing the probability of failures in early and late stages of the life cycle of a machine.

Figure 4 shows the probability distribution of failures over the life cycle of a machine. The risk of failure is higher at start-up, then drops and increases again with wear-out. The statistically determined period between these states can be used as a determinant of the replacement period [24,25].

The disadvantage is also the necessity of planning maintenance and costs. Effective preventive maintenance planning in energy generation should align maintenance intervals with the required plant availability. For the time-based approach, the authors of works [26,27,28,29] propose a cost-reliability model to find the optimal policy by improving reliability over low cost. Planning the schedules requires historical data for analyses of maintenance history, usage conditions or a failure history (we may use specification for the same or a similar device, alternatively, data from the manufacturer). An alternative group of methods relies on task planning with Key Performance Indicators (KPIs) [30,31,32] or actual condition monitoring [33,34,35].

### 2.3. Predictive Maintenance

In predictive maintenance, the servicing is carried out when it is required, usually shortly before a fault is expected. The essence of this approach is to predict the health of a machine based on repeated analysis or known characteristics. Therefore, predictive maintenance is a type of condition-based maintenance in which we predict future performance based on current and historical indicators. The application of this technique leads to a reduction in both planned and unplanned downtime. Planned downtime refers to preventive actions that can be better scheduled and unplanned downtime is related to unexpected failures that can be avoided by continuously monitoring the equipment condition.

Commonly used conventional predictive maintenance techniques are based on periodic measurements that cover the following [23,36].

#### 2.3.1. Vibration Monitoring

This technique applies to all motion and rotating machinery and is widely used in the industry for diagnostic, condition monitoring and prediction functions. Predictive techniques involve trend analysis for vibration levels, in particular frequency ranges or signal profile analysis. Trend analysis is used to determine remaining useful life and to evaluate component deterioration. Since the vibration level is itself an indicator of poor condition, predictive analyses can easily be made using only this measurement. Signal profile analysis provides the possibility to detect and classify unwanted events. Detection of characteristic signal patterns or anomalies enables discovering specific faults such as leaks, seizures or material loss. The use of this method is costly because it requires the installation of additional measurement equipment. However, developing analytical methods such as those based on machine learning allows for rapid diagnostics without involving experts in the analysis. The vibration monitoring can cover devices and their components such as pumps, fans, compressors, gearboxes, engines, turbines.

#### 2.3.2. Thermography

This technique is used to predict and diagnose the condition of equipment and systems based on temperature measurements. Advanced instrumentation allows for monitoring infrared emissions using thermal imaging cameras, infrared thermometers or line scanners. The analysis of obtained results (temperature, its variations and distribution) allows determining the condition of the device and detecting potential anomalies. In practice, thermography can be used as a non-destructive method to detect wall thickness caused by corrosion and flow erosion in high-temperature pressure pipe [37] and to determine the loss of material in the boiler water–wall tubing [38]. Thermography is also applied for diagnosing electrical equipment, detecting oil leaks [39] and detecting faults in photovoltaic (PV) farms. In the latter case, it enhances the capability and safety of inspections [40,41] and provides methods to determine PV panel health [42,43].

#### 2.3.3. Oil Analysis

Oil plays a vital role inside a working machine—it is responsible for lubricating, cooling, cleaning, protecting or sealing [44]. The systematic analysis of the chemistry and contamination of oil can provide indicators of the wear of machine components and lubrication quality. Systematic analysis of oil makes it possible to determine the state of wear of machine elements [45,46] and to plan preventive actions, such as changing oil or filters more effectively [47]. Investigations may include testing of viscosity, contamination, solid content, oxidation, nitration, total acid number, total base number, particle count. Examination of these properties can determine the quality of the lubricating performance, detecting leaks, corrosion or abnormal wear. Spectrography and ferrography are also complementary techniques in this area, allowing for the analysis of contaminants and additives. Using these methods, we can perform wear particle analysis to determine the types of deterioration such as rubbing wear, cutting wear, rolling fatigue and sliding wear [48,49]. Limitations of this method are the equipment cost and the difficulty in oil sampling and interpretation of results.

#### 2.3.4. Acoustic Analysis

This technique includes analysis of acoustic signals, noise and ultrasound. With relatively inexpensive tools, it is possible to monitor rotating machinery in a similar way to vibration analysis. By analyzing the signal in the frequency domain, we can detect anomalies caused by friction and stresses that may be symptoms of deterioration. In the case of the detection of gearboxes defects, acoustic analysis can complement vibration monitoring for the detection of more minor defects [50]. On the other hand, ultrasonic analysis is used to detect leaks in valves and pipes. Leaks generate an identifiable signature in the high-frequency band [51]. By investigating the shape and characteristics of the ultrasonic waveform, we can detect cavitation in the centrifugal pump [52].

#### 2.3.5. Motor Current Analysis

The electric motor is an integral part of most power plants. Its failures often lead to energy production outages. Therefore, it needs special attention. It is exposed to mechanical faults characteristic of rotating machinery, but a significant part of them is caused by electrical faults. Common failures include bearing failures, stator winding faults, rotor faults, insulation faults [53]. The methods used here (in addition to vibration and acoustic monitoring) cover:

- Insulation resistance test—insulation may be damaged by high temperature or can be contaminated by humidity. The test consists of grounding the motor frame and applying DC voltage to the motor windings with a measuring device. Then, the device reads the resistance value [54].
- Motor Current Signature Analysis—this is a technique used to analyze and monitor electrical induction motors, generators, power transformers and other electric equipment. This method uses the supply current to produce the current signature from frequency spectrum transformation. Faults in motor components produce anomalies in a magnetic field and change the mutual and self-inductance of the motor that appear in the motor supply current spectrum [55,56]. This method allows detecting faults such as [53,57]:
  – Broken Rotor Bar—a fault that can cause sparking and overheating in a motor. Examining the frequency spectrum of the stator currents can provide early fault detection [58,59].
  – Bearing Faults—faults caused by misalignment after bearing installation [60] and increased vibrations [61].
  – Eccentricity-related faults—a condition when air gap distance between the rotor and the stator is not equal [62,63].
  – Stator or Armature Faults—faults usually related to insulation failure. Shortened turns produce excessive heat in the stator coil and current imbalance [64,65].
  – Equipment wear—a degradation of parts observed in the long term. Equipment wear is also visible as changes in the current spectrum.

#### 2.3.6. Analysis of Process Parameters

This approach relies on actual measurements to determine indicators of process performance or health index. By monitoring the index over time, we can assess changes in the equipment condition. This technique is widely used in combination with the Internet of Things (IoT), machine learning and big data technologies. It is possible to utilize the enormous amounts of data generated in technological systems for predictive maintenance tasks. Performance indicators can be directly calculated based on the data. For example, we can calculate the efficiency of a pump based on the flow, heat and power by computing the ratio of output to input power [66]. Other ways are to create a health index model based on historical data or data from a similar machine [67].

#### 2.3.7. Visual Inspection

Online condition monitoring and predictive maintenance improvements sometimes cannot replace traditional inspection methods. To avoid undetected faults, maintenance with defined models and installed metering should be supported by engineering experience in the inspection process. The traditional process can be supported by modern technologies that enable mobility and access to information. Inspections are supported by augmented reality [68], mobile applications [69], radio-frequency identification (RFID) [70] and barcodes.

## 3. Techniques and Methods in the Maintenance Area

Decisions regarding which equipment to include in which strategy, how to schedule maintenance and how to manage materials are based on the adopted asset-management methodology and assumptions regarding expected availability and efficiency. The outlined processes and techniques allow for continuous improvement and achieving the objectives. The effects of their implementation are driven by control, documentation and integration of data sources related to the history of equipment repairs, diagnostics, warranties or regulatory recommendations. Typically, these data are stored in the enterprise asset-management (EAM) system or computerized maintenance management system (CMMS). An additional advantage is the use of process data (from operational technology) to determine performance and health indicators.

### 3.1. Total Productive Maintenance

Total Productive Maintenance (TPM) is an employee-focused methodology that concentrates on continuous improvement of equipment effectiveness by involving all employees in maintenance tasks, training the staff and ensuring proper communication between operators and technicians. TPM makes efforts to eliminate the following losses [71]:

- Breakdowns;
- Setup and adjustment;
- Idling and minor stoppages;
- Reduced speed;
- Defects in a process;
- Reduced yield.

A metric for TPM performance is the overall equipment effectiveness (OEE) factor, which is the multiple of availability, performance efficiency and quality rate. TPM derives from other methods of lean manufacturing, such as 5S, 5WHYs or kaizen, but the following activities are specified as being essential:

- Education and training;
- Autonomous maintenance;
- Preventive maintenance;
- Planning and scheduling;
- Reliability engineering and predictive maintenance,
- Equipment design and start-up management.

### 3.2. Reliability-Centered Maintenance

Reliability-Centered Maintenance (RCM) is defined as a process used to determine what must be done to ensure that any physical asset continues to do what its users want it to do in its present operating context [72]. The primary purpose of RCM is to preserve the system functions rather than to keep the asset in service. The implementation of RCM requires a complete understanding of the functions of the physical asset and the nature of failures associated with those functions. Due to the individual treatment of each type of failure, it may overlook some events that could affect, for example, life expectancy or performance loss [73]. RCM focuses on finding answers to the following questions:

- What are the functions and associated desired performance standards of the asset in its present operating context (functions)?
- In what ways can the asset fail to fulfill its functions (functional failures)?
- What causes each functional failure (failure modes)?
- What happens when each failure occurs (failure effects)?
- In what way does each failure matter (failure consequences)?
- What should be done to predict or prevent each failure (proactive tasks and task intervals)?
- What should be done if a suitable proactive task cannot be found (default actions)?

RCM can be successfully applied to maintenance management in power distribution. Authors of the work [74], by including the downtime cost of outages, designed an optimization algorithm that allowed to reduce total costs while increasing reliability level. A comprehensive framework to implement RCM, including cost–benefit ratio for critical power distribution equipment, was investigated in Sweden [75]. The analysis of RCM application carried out for important elements of wind turbines was reported in [76], where the authors drew attention to the importance of the standardized and automated collection of failure and maintenance data.

### 3.3. Failure Mode and Effect Analysis

**Figure 5:**
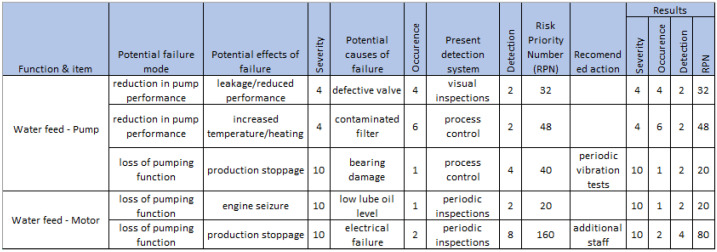
Example of the FMEA sheet.

Failure Mode and Effect Analysis (FMEA) is a methodology to identify potential failure modes for equipment or process, evaluate the risks associated with them, prioritize problems, and identify and execute corrective actions to resolve the most significant problems. Failure mode effects analysis and reliability-centered maintenance are important methods for implementing preventive maintenance programs [77]. Depending on the stage of development, the following types of FMEAs are distinguished into those focusing on System, Project, Process or Service. FMEA analysis consists of a functional decomposition of the object or process being investigated and the collective group of quantitative and qualitative analysis data [78]. An example of the FMEA sheet is shown in Figure 5. It consists of the following elements:

- Item;
- Function;
- Failure;
- Effects of Failure;
- Causes of Failure;
- Assessment rating;
- Recommended Action.

Qualitative analysis is used to evaluate risk and prioritize corrective actions. It focuses on possible defects, their causes and their effects. Quantitative analysis includes a criticality analysis for each component at a given operating time and identifies the component reliability associated with each potential failure mode. For each failure mode, it also evaluates the probability that the component will cause a system failure.

To evaluate the impact of the identified defects, we can use the risk priority numbers (RPN) method. The method involves determining the following:

- **Severity** of each failure;
- Likelihood of **occurrence**;
- Difficulty of **detection**.

The *RPN* is then calculated as:

RPN=Severity×Occurrence×Detection

FMEA analysis was successfully applied to a wind turbine assembly reported in [79]. The authors compared quantitative FMEA results with field failure rates showing meaningful similarity. The results obtained are useful for the future design of wind turbines. Furthermore, the paper points to software supporting FMEA analysis [80,81].

The execution of FMEA analysis can be easily digitized and used in a semi-automatic mode using information systems. Several papers, including [82,83,84], propose coding the FMEA structure using a Bayesian network. The inclusion of probabilistic data allows for better risk analysis and provides opportunities for further insight processing.

The extension of failure analysis by using data-driven methods is presented for actual data in a hydroelectric power plant case study presented in [85]. The authors extended the fundamental FMEA analysis with Association Rule Mining and Social Network Analysis.

Moreover, many works are devoted to the automatic generation of FMEA surveys by using historical data and simulation methods that allow this process to be carried out without involving experts [86,87,88]. An example of the application of a data-driven method using a deep learning technique for the aviation industry has shown that the accuracy of the fault prediction can oscillate around 95% [89].

### 3.4. Fault Tree Analysis

**Figure 6:**
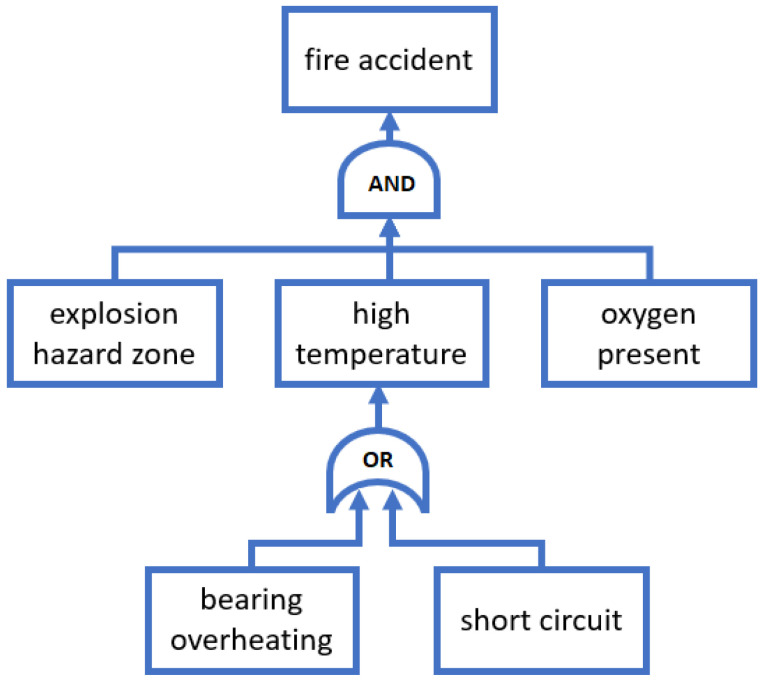
Example of fault tree analysis for fire accident.

Fault tree analysis (FTA) is a technique that uses a graphical and logical model to describe the relationships of multiple events leading to a failure. A top-down diagram consists of two types of elements: events and logic gates that link to identify the cause of the top undesired event, as shown in Figure 6. The main goal of FTA is to reduce the risk of system failure based on the identified causes and their probabilities. In addition, through this analysis, we can achieve improved system performance. FTA can be performed standalone or as a complement to FMEA. The main benefits of FTA are:

- Graphical visualization;
- Support in identifying the reliability of single components or the whole system;
- Support in determining the probability of occurrence for each root cause;
- Assessment of the impact and risk of possible changes;
- Capability to highlight the critical components;
- Identification of paths leading to failures;
- Capability to perform qualitative and quantitative analysis.

Examples of the use of FTA analysis in the industry include fault tree analysis for power transformer based on analytical methods and infrared diagnosis [90] or calculation of fire and explosion risk using expert knowledge and probabilistic methods [91]. The development process is supported by tools that facilitate fault tree diagram generation. Semi-automatic building is based on translating a model written in SysML [92], UML [93], AADL [94] or is generated from a defined set of components, function tables and transition tables [95,96].

### 3.5. Root Cause Analysis

**Figure 7:**
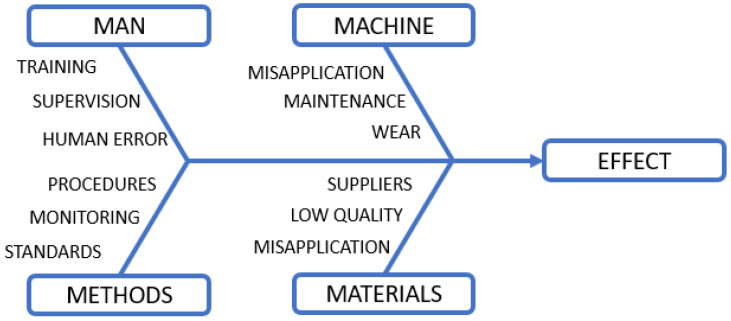
A fishbone diagram with various categories of failure or defect cause [97].

Root cause analysis (RCA) is a technique that performs deep analysis to identify the underlying root causes of failures to avoid their future occurrence. In the literature, we can also find the RCA under synonymous names *fault isolation* or *fault localization*. The process is widely used in industry due to the complexity of systems where associating faults with their symptoms is not always trivial. For example, in the case of a seized bearing, the corrective approach is limited to replacing the component with a new one. At the same time, root cause analysis focuses on finding the root cause, e.g., poor lubrication, oil leakage, vibration, etc., which will help avoid future incidents. Classical inference methods rely on deductive, knowledge-based analysis where we can use the following techniques:

- 5WHYs is a deductive method that involves iterative asking of “why” questions for the failure that has occurred. With the correct formulation of the questions and maintaining the cause–effect logic, the method allows the analysis of the source of the defect and learning more about its causes.
- Fishbone diagram, also called Ishikawa diagram, is used to visualize cause-and-effect relationships, thus helping to distinguish the causes from the effects of a particular failure and perceive the complexity of the problem. The analysis starts with determining the occurrence of the event (failure or defect) and proceeds to identify all possible factors that caused it while categorizing the groups of the causes. A typical diagram divided into 4M categories (Man, Machine, Methods, Materials) is shown in Figure 7.
- A Pareto chart, which is a simple tool that easily categorizes and visualizes data in a bar chart. By following the 80/20 rule (20% of causes cause 80% of problems), the method allows highlighting those causes that provide the most substantial quantitative or financial impacts.

Manual analysis by experts is usually of very good quality. Still, it is very time-consuming and requires extensive industry knowledge, which, together with a complex system, limits the applicability of the method. Therefore, it is worth considering the use of data-driven algorithms to automate the process or assist in making it significantly more straightforward. The used techniques inherited features from areas such as Artificial Intelligence, neural networks, graph theory, statistics and automata theory. While analyzing various algorithms used for root cause analysis, Solè et al. [11] categorized them as:

- Deterministic—based on designed rules, including such implementations as fault tree, codebooks, Petri nets.
- Probabilistic—dealing with the uncertainty issue and involving stochastic methods, including Bayesian networks, hidden Markov models, decision trees or fuzzy logic.

Analysis models may have different properties and performances that affect the purpose of using them. The purpose of the analysis may be to detect root cause based on symptoms, with algorithms capable of detecting, classifying or multi-classifying events occurring simultaneously. The purpose of the analysis may be just to detect or classify root causes based on symptoms or to drill down to explain their nature, origins and related incidents fully. Thanks to this, the algorithms may have the capability to detect, classify or multi-classify events occurring at the same time.

## 4. Value of Advanced Analysis in Maintenance

This chapter will provide examples of how we can use data and analytical opportunities to derive value in the area of maintenance. The methods presented here, categorized in terms of complexity and purpose, allow describing past events and the current situation accurately, predicting outcomes in the future and simulating and optimizing expected results.

Section 4.1 presents a general categorization of approaches at a high level of abstraction according to the scope of analysis and the methods used. Subsequent sections address the issues of prognostics and health management. Section 4.2 presents a categorization of the described PHM methods. Section 4.3, Section 4.4 and Section 4.6 describe in detail three groups of methods—model-based, data-driven and signal-based. Section 4.7 is devoted to the area of determining the remaining useful life. Section 4.8 briefly presents methods from the prescriptive area, including applications of simulation, digital twins and advanced optimization methods.

### 4.1. Complexity and Scope of Analysis

Digitization of datasets and the capability of real-time processing allow the use of automation and inference to support traditional maintenance processes. Implementation of data mining systems gives undoubted benefits to the process but also requires the involvement of particular resources and, depending on the issue being solved, necessitates engaging in varying levels:

- IT infrastructure (data repositories, cloud, interfaces);
- Expert knowledge (domain knowledge and data science);
- More or less availability of historical data.

**Figure 8:**
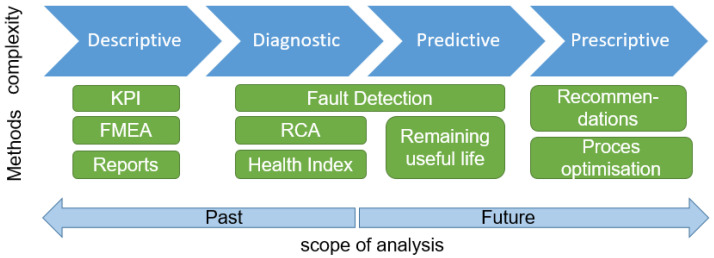
Scope of insights in analysis.

Depending on the complexity (and related uncertainty) of the analysis and the time context of the result, the following types of analysis can be distinguished, as shown in Figure 8:

- Descriptive—answering the question “what happened?” This type of analysis is used to interpret historical data to understand the process better and determine metrics to evaluate and compare performance. An example would be calculating the MTTF (mean time to failure) for a system component. It involves tools and elements, such as reports, metrics, KPIs or graphs, mainly using statistical methods and data visualization.
- Diagnostic—answering the question “why did it happen?” This type of analysis relies on data-mining techniques to determine the current state and/or its causes. In maintenance tasks, the multi-level analysis includes:
  – Fault detection: online detection of a fault or anomaly condition, determining current health index;
  – Fault isolation: determination of failure location;
  – Fault identification: categorization of the fault.
- Predictive—answering the question “what will happen?”. This type of analysis utilizes statistical tools and machine learning to predict future conditions. In the context of the research area, it is related to the introduction of predictive maintenance techniques, enhancing the opportunities by using new information technologies that allow online data acquisition, integration and analysis. Predictive techniques focus on detecting future failures and determining the remaining useful life indicators, providing the expected time to failure.
- Prescriptive—simulates possible scenarios for different decision paths based on the prediction results and chooses the optimal solution according to the assigned target function. This type of analysis engages Artificial Intelligence, optimization and simulation techniques to support real-time decision-making. An example could be an algorithm that controls the operation of a machine in such a way as to extend its remaining useful life to the nearest planned downtime.

The following sections detail the predictive algorithms categorizing and extensively describing the prognostic and health-management methods.

### 4.2. Classification of Prognostic and Health-Monitoring Methods

**Figure 9:**
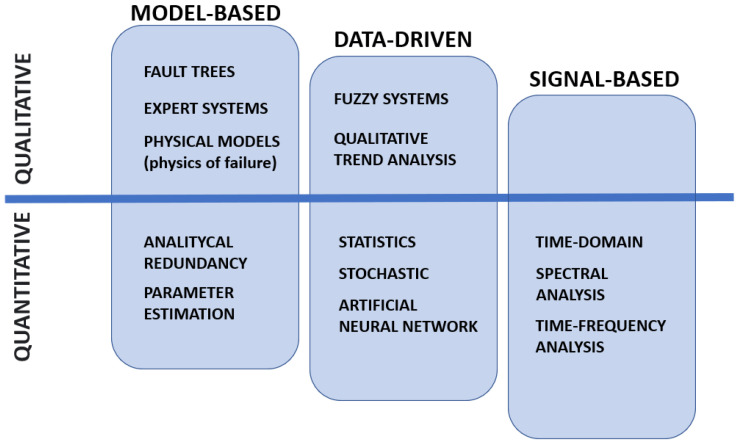
Categories of failure detection and identification methods.

Failure detection and diagnostics methods can be classified in different ways considering the used approach, employed resources or techniques. Most commonly, we can group the methods as data-driven and model-based. Some authors also distinguish subgroups sich as knowledge-based, signal-based or stochastic. A hybrid approach is also often used as a combination of several defined methods. According to the taxonomy adopted in the literature and considering the characteristics of the described models, we propose a two-dimensional classification shown in Figure 9. The methods are mainly categorized as:

- Model-based—a group of model-based approaches requires the system to be designed so that the expert knowledge is encoded in such a way that we can automate the diagnostic process. The model is designed in a deterministic way, e.g., using equations and mathematical modeling, together with flows or graphs to replicate the behavior of the system. These approaches are sometimes referred to as white boxes, where the relationships between inputs and outputs are carefully designed and predictable.
- Data-driven—approaches that use historical datasets and techniques such as machine learning to create inference rules. In general, data-driven approaches generate a model called a black-box due to limited insight into the structure and mechanics of the model. The design process is based on careful selection of training data and the choice of an appropriate architecture/technique. It requires much less domain knowledge at the cost of slightly more input from the data scientist.
- Signal-based—approaches that are very similar to traditionally used diagnostic methods. They are based on the assumption that the measured signal reflects the fault condition. The used techniques rely on studying a single signal/measurement, with feature extraction and decomposition of the measured value being the main elements.
- Quantitative—quantitative approaches focus on determining the relationship between the input and output of the system. They concentrate on heuristics using mathematics, statistics and stochastics and taking into consideration the potential uncertainty.
- Qualitative—in qualitative models, relations in the system are expressed by qualitative functions of specific system parts, formulated as casual graphs or IF–THEN rules.

Section 4.3, Section 4.4 and Section 4.7 describe in detail the methods included in the categorized approaches: model-based, data-driven and signal-based.

### 4.3. Model-Based Methods

Model-based methods draw from expert knowledge, making the created system transparent, clear and well-reflecting with respect to the state of knowledge. Here we present the processes that belong to this category together with their description and examples of application in fault-detection and -identification tasks.

#### 4.3.1. Fault Trees

A fault tree represents a structure of the cause-and-effect process in the form of a visual diagram. The application of this method is user-friendly and transparent. It provides an opportunity to describe events (e.g., valve blockage, short circuit) that are difficult to represent with quantitative methods based on measurements [98].

Fault trees have been successfully used in the area of reliability and safety assurance—Purba [99] presents the application fault trees in combination with a fuzzy-based reliability approach to calculate the probability of basic events. The quantitative complementation of the fault tree overcomes limitations caused by a shortage of fault probability distributions. Fault trees extended by fuzzy set theory have also been employed in the petrochemical [100] and mining industries [101] to manage the risks associated with fires and explosions.

Traditional root cause analysis using fault trees and fish-bone diagrams was applied to a gas turbine in a power plant. By considering probabilities and potential costs, Sarkar et al. [102] were able to achieve design improvements and support scheduled maintenance planning.

#### 4.3.2. Expert Systems

**Figure 10:**
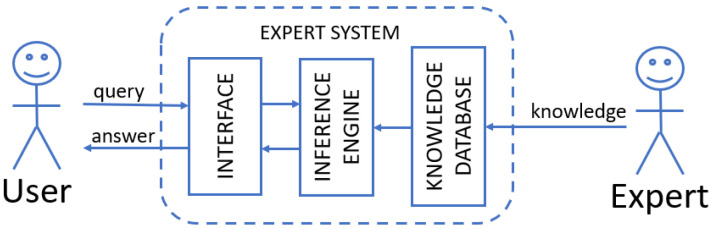
Illustrative schema of the expert system.

The idea of an expert system is to encode the knowledge of industry experts in the form of an algorithm so that the inference process can be automated, e.g., by a computer program. The schematic diagram of the expert system is shown in Figure 10. The user interacts with the system via an interface. It can be a typical computer application, but recently many approaches have used mobility, augmented reality [103,104] or virtual assistant [105]. The knowledge database is a structured repository of documentation, solutions and experience, powered and continually updated by domain specialists.

Based on reviews of available techniques [106,107], we can distinguish the following main techniques applied in expert systems:

- Rule-based systems—these involve encoding the operation logic in terms of IF–THEN expressions. The technique allows codification of the records of operating instructions, e.g., IF *the bearing temperature exceeds a predetermined threshold*, THEN *send an alert*.
- Case-based reasoning (CBR)—this involves the use of a type of knowledge database that, relying on similar preceding cases, provides a solution for the current problem.
- Neural networks and evolutionary algorithms—these are two soft computing approaches that provide algorithms based on, for example, artificial neurons or genetic algorithms [108], instead of mathematical logic, for the inference step.
- Fuzzy system—this is another soft computing approach that relies on fuzzy set theory and allows incorporating uncertainty into the inference. It uses statistical and probabilistic methods to reflect human-like decision-making. Compared to the standard IF–THEN rules, such as the following one:
  **IF**  *T*  >  *70*  **THEN**  *stop*
  fuzzy rules contain premises and consequents that are based on fuzzy sets:
  IF (T is High) and (T is rising) THEN stop
- Object-oriented methodology—this focuses on storing procedures and data in the form of classes and hierarchies. Objects (instances of classes) store values, text, graphics, diagrams and all functional information. The method allows modeling facts and relationships using three concepts: abstract data typing, inheritance and object identity.

#### 4.3.3. Analytical Redundancy

**Figure 11:**
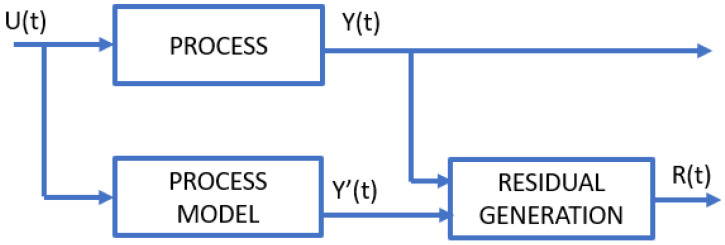
Analytical redundancy and residual generation.

One of the most commonly used model-based fault/anomaly detection methods involves analytical redundancy [109]. The method consists of modeling the investigated signal or process (U) and examining the difference between the actual output value (Y) and the estimated one (Y’). The resulting difference, also named residual (R), is analyzed in the next step for fault diagnosis. A simplified flow within this method is shown in Figure 11.

The model may be a physical copy of the device (hardware redundancy) or a mathematical expression (either deterministic or statistical) that describes the investigated value based on the relationships between process variables. Depending on the underlying dataset, there are two types of redundancy. Direct redundancy analyzes relationships between correlated instantaneous sensor outputs, while temporal redundancy estimates values based on both sensor outputs and actuator inputs over a time span.

Within this approach, three groups of techniques are distinguished:

- Observer-based—this relies on comparing the measured value with the estimated one for each particular signal. Internal states are represented by the relation between input and output. In the healthy system, residuals should oscillate around zero, while significant values should indicate failure states. The methods within this group were originally based on the Luenberger observer [110,111] or Kalman filter [112,113]. Recent work describes novel observer-based methods for distributed fault estimation in complex multiagent systems with nonlinear dynamics. Liu et al. [114] present a fault-detection method treating a fault as a special state of the system and using the outputs of neighbor nodes to estimate the fault state by the observer. Han et al. [115] propose a method for a topology defined by a directed graph, where using Schur decomposition makes the system computationally efficient regardless of the number of nodes.
- Parameter estimation—this assumes that the fault affects the system parameters (which are not necessarily measurements). The technique involves examining changes in the estimated parameters in the continuous domain, e.g., by comparing them to a model condition for the healthy system or checking changes in characteristics [116].
- Parity space—this is a technique similar to the observer-based one, but the essence is to obtain residuals vector (parity space, residual space) by comparing the consistency of results generated by digital models with measurements (sensor outputs) or process inputs (actuators) [117,118]. Fault identification for sensors can be accomplished by designing relationships so that the values of individual residues are associated with specific sensors. Similarly, for actuator fault identification, we can use parity space transformations so that non-zero results clearly indicate the source of the fault. Examples of such an approach are the following: single actuator parity relation [119] or orthogonal parity equations [120,121].

### 4.4. Data-Driven Methods

Data-driven methods use available data resources and extensive historical data repositories to model processes and provide inference engines. The methods presented in this group use advanced analytics, including techniques such as machine learning. The main drivers here are the quality of the training data, the ability to process large amounts of data, including streaming data and analytical skills supported by domain knowledge. Important factors determining the quality of the results obtained are quality of training data, data processing capabilities and analytical knowledge.

The spectrum of methods used in this category cover the use of:

#### 4.4.1. Fuzzy Systems

Fuzzy logic-based techniques are often categorized as expert systems, with the added advantage of quantitative rules that improve efficiency in dynamic systems and situations of uncertainty. The essence of fuzzy systems is to transform a vector of input data into a fuzzy set and apply fuzzy rules to inference, which is similar to human reasoning [10]. This means that discrete inputs such as “0” and “1” can be evaluated with respect to intermediate states according to a rule that takes into account the influence of other variables or the change history. Designing the fuzzy system requires including expert knowledge for the fuzzy rules that are created to improve system performance. Knowledge can be drawn from both expert experience and historical data, so the technique is often used in conjunction with expert systems or artificial neural networks.

Fuzzy logic has been applied in instrumentation monitoring, enhancing the operator’s perception and decision-making on the system’s condition and the potential maintenance tasks [122].

#### 4.4.2. Qualitative Trend Analysis (QTA)

**Figure 12:**
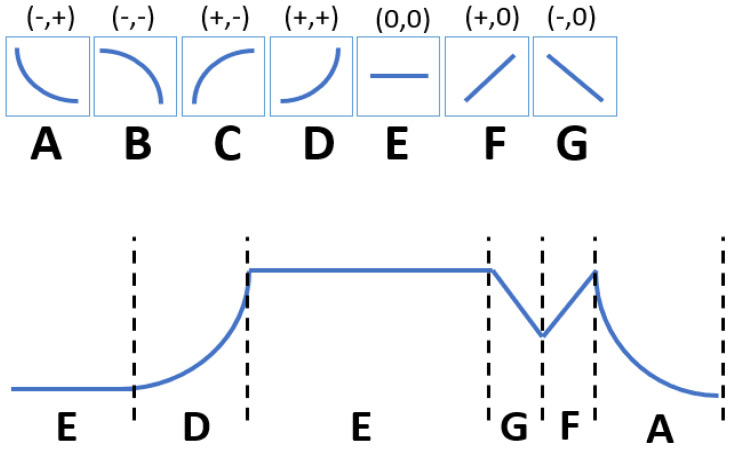
Example of time series segmentation.

This technique relies on series analysis by extracting qualitative features from observed trends. In most cases, the sequence of identified shapes of measured signals reflects significant events affecting process behavior. The technique involves segmenting the time series into episodes so that the end of one is the beginning of the next. Each episode has a qualitative state dependent on the derivative of the signal: increasing (+), decreasing (−) or constant (0) [123]. An example of segmentation is shown in Figure 12. The analysis with these methods consists of two steps:

- Trend extraction—a transformation of the data series into trend patterns. Here we can use methods such as wavelet transform [124], neural networks [125] or hidden Markov models [126,127].
- Trend analysis—based on the characteristics of the trends sequences, the qualitative features are obtained to classify characteristic events.

This group of methods is used to assist the operator in analyzing process data, automatically detecting fluctuations before a failure occurs [128], but can also be used to monitor the effectiveness of a process control loop [129].

#### 4.4.3. Statistical Methods

In fault-detection and -diagnosis tasks, quantitative statistical approaches focus on pattern recognition based on features extracted via statistical methods. Widely used statistical fault-diagnosis techniques include principal component analysis (PCA), partial least squares (PLS) auto-regression methods and Support Vector Machines (SVMs).

- PCA— thisis one of the most commonly used techniques; it transforms the input data vector to reduce its dimensionality with minimal loss of information. The transformation provides fewer features to represent system characteristics, trends and states, simplifying the analysis and further computations. PCA-based methods have been applied recently in several power plants, improving the diagnostic potential and reducing the number of false alarms [130,131,132].
- PLS—this is a statistical method that finds relationships between features in a linear regression model projected onto a new projection space. The PLS and PCA techniques were used in detecting coal mill blockages [133]. PLS was also applied in the performance monitoring of power plants. By using historical data from process control systems, it is possible to estimate the efficiency of the gas turbine [134] or quality measures of thermal efficiency and NOx and SOx emissions [135].
- SVM—this is a supervised learning technique based on statistical learning theory [136] that can be applied to both classification and regression tasks. The method involves finding a decision boundary in a space, using a transformation function (named *kernel*) that maps examples between two classes with a maximum margin. It is widely used in fault detection, typically for equipment in thermal power plants [137] or wind turbines (with comparable accuracy to artificial neural networks) [138].

#### 4.4.4. Stochastic Methods

The stochastic approach includes quantitative methods that create conditional probabilistic models. This group comprises algorithms that diagnose and predict the states (failure and failure-free) defined based on measurement indications and derived probabilities. It generally uses Bayes’ theorem, where the conditional probabilities *P* of two different events, *A* and *B*, are determined according to the equation:

$$P\left(A|B\right)=\frac{P(B|A)*P(A)}{P(B)}$$

A Bayesian network is a representation of a system using a directed graph with nodes representing random variables and states that reflect a cause-and-effect relationship for predicted failures [139]. Despite being categorized as data-driven, stochastic algorithms do not require an extensive and complete dataset. A representative subset is sufficient to determine the probability distribution. The structures and algorithms are very transparent. However, they often require qualitative work in the form of FMEA or RCA analyses to create the necessary structures.

Methods based on Bayesian networks are often combined with other methods to increase the quality of calculations. For example, these can be:

- Particle filters—this method uses a partial dataset to estimate the state of the system. It is fed with random samples (particles) migrated into groups to estimate posterior distribution [140]. The method can be used to model nonlinear system characteristics with various types of noise.
- Kalman filter—this is a state estimator that operates on a dynamic system with a Gaussian noise distribution. The state is estimated from a series of current observations (could be incomplete) and the recent system state. It is a computationally efficient algorithm, mainly when applied to linear systems. The Extended Kalman Filter is successfully used with nonlinear input–output relationships.
- Markov models—Markov models represent a system where the individual states reflect observable events or conditions. The predicted state depends on the sequence of previous states. The extension of the models are Hidden Markov Models, where the process is coded in terms of hidden chains, in case the model is not trivial to describe. With the Markov models, we can model both spatial and temporal events. The disadvantage of the method is the high computational complexity.

#### 4.4.5. Artificial Neural Networks

Artificial neural networks (ANNs) [141] have many application scenarios, including fault-detection and -diagnosis tasks. These types of networks can be categorized based on the architecture adopted and the output goal designated. The method relies on machine learning, which involves adjusting the parameters/weights of a neural network in a sequential training process based on a large amount of historical data. Creating a model does not require engaging an expert’s knowledge but instead needs having a substantial dataset and requires performing the learning process appropriately. The learning strategy is categorized as supervised and unsupervised. Unsupervised learning adjusts the network to the training data, e.g., the network is adjusted for a failure-free period and tracks deviations from this state for production work, or time series are continuously analyzed looking for deviations. Supervised learning requires labels to be specified in the training set and usually needs more input in data pre-processing.

We can categorize the types of ANNs based on the architecture adopted, output goal and learning method.

Considering the output values, the ANNs may perform the following tasks:

- Detection—when the output is a binary true/false value. They are used when detecting specific events [142,143] or anomalies [144,145].
- Classification—the process categorizes the data in a way that reflects the relationship in the training set by assigning the defined label (specific fault, state of health). An example is the classification of a fault condition in a wind turbine [146].
- Regression—this generates a continuous value at the output, usually a residuum or a measure of device health. The method has a wide range of applications, from trend analysis to remaining useful life predictions.
- Clustering—this is a method that uses unsupervised learning, i.e., the training set requires no labeling. On the basis of the training set, the data are grouped and/or prioritized. The method is used in particular for detecting anomalies. For example, Rakhshani et al. [147] group boiler health states in a power plant and use ANNs for failure prediction.

The classic neural network architecture is a feed-forward network, such as MLP (multi-layer perception) or RBF (radial basis function). The networks consist of an input layer, optional hidden layers and an output layer. The input data can be both quantitative and qualitative and the input dataset is often processed through a feature selection algorithm (to extract the most relevant features) or dimensionality reduction (e.g., using principal component analysis [148]). The feature selection process is properly solved for most deep learning architectures, i.e., having more complex schema and hidden layers. An example is the use of a Convolutional Neural Network (CNN) autonomously performing feature engineering. Janssens et al. [149] obtained much better results with CNN than with the random forest with manual feature engineering, showing time savings on data pre-processing.

A frequently used model, especially in anomaly detection, is auto-encoder. The principle of the auto-encoder is to learn the system representation in an unsupervised way by reconstructing the output of the input signal through lossy compression in the hidden values. The natural application of the network is signal denoising [150], which makes it useful as a pre-processing step in many systems [151]. It can also be used to detect anomalies by generating residuals from differences between the input and output vectors [152,153,154,155,156].

A noteworthy architecture in the area of fault detection and identification is proposed in recurrent neural networks (RNN). A characteristic feature of the RNN is backward connections from further layers, not present in the feed-forward networks. This type of network is designed to analyze sequential data in time series. A sub-type of these types of deep networks is a *d* long short-term memory (LSTM) that allows for long-term time series analysis through the use of forget gates. The technique can be successfully used to detect failures of rotating machinery such as bearings [157], motors [158] or wind turbines [159].

### 4.5. Signal-Based Models

**Figure 13:**
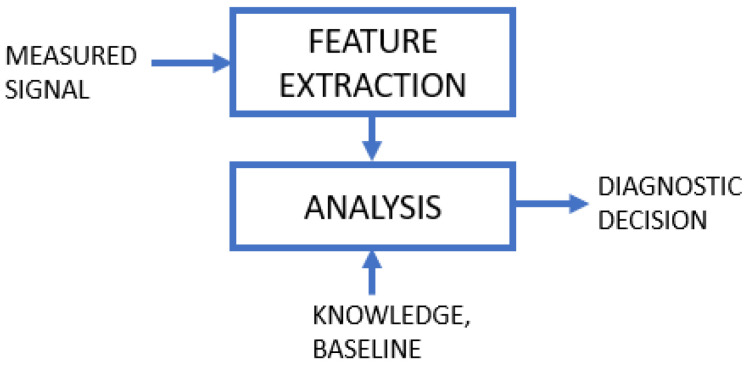
Signal-based failure detection and identification.

Signal-based models investigate the characteristics of the measured signal and compare them with baseline performance obtained for a healthy system or monitor its changes over time. A simple scheme of signal-based failure detection and identification is shown in Figure 13. Potential faults are reflected in the time series of the measured signals or their spectrum, allowing both the anomaly/fault detection and classification of specific faults. The method is widely used in traditional monitoring and diagnostics, investigating vibrations [160], acoustic or current signals [60,161].

Depending on the applied signal processing algorithms and extracted features, we can distinguish the most representative groups of methods, such as time-domain, frequency-domain and time-frequency domain. An extensive review of techniques in this category is described in articles [9,162], while here we present a brief summary.

#### 4.5.1. Time Domain (Temporal Analysis)

These methods operate directly on raw measured signals, exploring averages, trends, min/max values, standard deviation, etc. Within this group, we find techniques related to time series analysis such as CUSUM (Cumulative Sum), Exponentially Weighted Moving Average [163], auto-regressive fitting or root mean square error tracking [164]. This approach aims to determine statistical indicators that allow comparing current readings with a baseline condition.

The plenary gearbox fault-detection example uses the analysis of statistical indices such as root mean square and sample entropy considering the signal selection stage [165]. The root mean square coefficient is often used in time-domain methods as a measure of vibration energy, which directly bears the condition of a device.

The definite advantages of these methods are the ability to evaluate system degradation directly, ease of comparison and transparency. More complex tasks such as determining the source of an anomaly or classifying a fault require other methods, e.g., based on frequency analysis.

#### 4.5.2. Frequency Domain

This is a popular group of diagnostic methods for rotating and electrical equipment. They represent waveforms in the frequency domain and are based on identifying frequencies at which typical defects occur. The signal spectrum can be obtained using the fast Fourier transform, which decomposes the waveform into a sum of sinusoids of different frequencies. Vibration is the most common measurement for bearings and gearboxes, whereas, for motors, we can use methods of motor-current signature analysis (MCSA) [53].

An example of the use of spectrum analysis is the vibration analysis for gearbox bearings [166], which allows tracking the progress of the degradation state; the results obtained were correlated with the findings of periodic inspections.

In the case of electrical signal analysis, current frequency analysis and amplitude demodulation has been used to detect bearing faults in wind turbines [167]. The alternative use of the electric current analysis provides an additional economic advantage in the absence of online vibration monitoring.

The main advantage of frequency analysis is its capability to locate the degraded component of the system. However, the main drawback is its incapacity to identify the origin of the degradation when the system is not stationary. This implies the use of time-frequency analysis.

#### 4.5.3. Time-Frequency Domain

Time-frequency analysis provides feature extraction in both the temporal and spectral domains. It uses transformation tools to decompose the signal and extract feature information contained in non-stationary signals [168]. We can use the following techniques from this group in the maintenance area:

- Short-time Fourier Transform (STFT): this provides information in both the time and spectral domains by tracking frequency changes as a function of time. However, the calculation of the Fourier transform over successive time intervals makes this method computationally complex. Using the coefficients extracted via the STFT method, Cocconcelli et al. [169] proposed a simple decision rule to detect bearing faults.
- Wigner–Ville Distribution (WVD): this provides better time-frequency resolution at a lower computational cost. The disadvantage of the method is the occurrence of cross-term interference, which makes the interpretation of results difficult.
- Wavelet transforms (WTs): these provide powerful signal-processing methods, and are also often used in fault detection [170,171]. WTs evolved from the classical continuous wavelet transform (CWT) and discrete wavelet transform (DWT) approaches. Advantages of using WTs include obtaining high adaptive resolution and handling non-stationary signals. However, choosing the proper base function can sometimes be a challenge.
- Hilbert–Huang transform (HHT): this consists in decomposing the signal according to the empirical mode decomposition (EMD) methodology into so-called intrinsic mode functions (IMFs) and then the Hilbert spectrum is obtained. HHT is the most adaptive method for non-stationary and non-linear signals.

### 4.6. RUL—Remaining Useful Life

**Figure 14:**
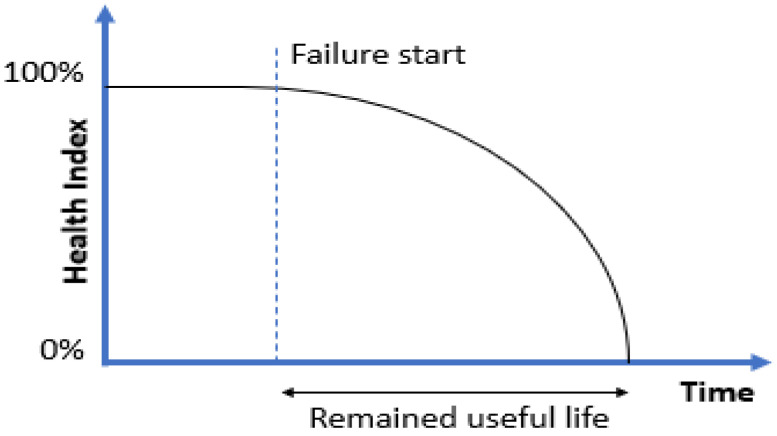
Remaining useful life based on equipment health.

The prognostic health-monitoring methods described previously can detect failure states and determine the health state of a device. An enhancement of the analysis carried out with these methods is to predict the exact time of expected failure and the degradation characteristics of machine health. The RUL (remaining useful life) indicator measures the time from the current moment to the estimated overall loss of functionality due to a failure, as shown in Figure 14.

The RUL time can be estimated based on one or more health (or degradation) indexes and/or investigating their variation over time. Many methods and algorithms used here coincide with those used in diagnostics and fault detection. The main difference is that in the RUL case, events are predicted in the future, which requires the adoption of certain confidence intervals and uncertainties [172,173]. The quality of a given health index and thus the applicability for the RUL task is determined by maintaining the following features and properties [174,175]:

- Monotonicity: this is the capability to maintain a constant increase or decrease in the health index over successive cycles, e.g., progressive wear in the absence of maintenance and continuous operation.
- Robustness: this determines the capability of the metric to make an appropriate prediction given the existence of noise and the degree of uncertainty in the results. Robustness results in a smoothed RUL characteristic.
- Trendability: this measures the correlation between the degradation rate and time.
- Identifiability: this enables classification of health status or failure modes on the basis of health index characteristics.
- Consistency: this is a ratio of the consistency of health indexes obtained with different methods.

Calculating the estimated remaining useful life requires comprehensive data on the current condition of the device and classification of potential failure modes. By correctly identifying the event that initiates a particular failure, we can determine the remaining useful life by extrapolating the degree of degradation and calculating the time when the threshold is exceeded. Alternatively, we can use historical records from the device and take into account the time decay of the health index for the “run to failure” operation. Thus, the most straightforward approach assumes the creation of degradation models for individual failure modes, identification of the current health index and/or failure modes, estimation of the remaining useful life by compiling information about the current state in the context of a probability density function for specific degradation cycles.

The classification of techniques proposed in the works of Sikorska et al. [8] and Lei et al. [174] includes the following approaches:

- Knowledge-based: Methods based on domain expertise, historical datasets and computerization that allows coding of knowledge, e.g., in the form of algorithms or rules in expert systems. These types of systems are easy to understand and design. The limitations are the functionalities determined by the knowledge of experts and the effort put into the design of the system.
- Statistical and stochastic methods: Statistical methods rely on analyzing current and past observations to predict future states. Methods are most often based on time series analysis and do not require large amounts of historical data. Distinctive methods used in this area include:
  – Autoregressive models allow estimating a parameter correlated with the RUL through time series analysis. The models used assume monotonicity and linearity of the estimated value concerning past data. The techniques used in this area are mostly based on moving average: ARMA (autoregressive–moving average), ARIMA (sutoregressive integrated moving average), WMA (weighted moving average) or ARMAX (autoregressive moving average with ecogenous input).
  – Markov models: these assume that machine degradation processes are contained in finite state space. By defining the probabilities connecting the different states, we can estimate the probability of predicted events such as a failure. The prediction values depend on the sequence of the last states in the analyzed time series.
  – Proportional hazard models (PHMs): these rely on the survival model proposed by Cox [176]. The PHM takes into account the influence of many factors on the estimated outcome. The main components of the factors of hazard function λ(t) are the following: baseline hazard function $\lambda_{0}$, which describes the degree of degradation in subsequent cycles; and covariate function exp(β×X), which describes the impact (β) on the hazard rate of the occurrence of conditional events (X):
    $$\lambda \left(t\right)=\lambda_{0}\left(t\right)\times exp\left(\beta_{1}X_{1}+\beta_{1}X_{2}+\ldots +\beta_{n}X_{n}\right)$$
- Machine learning (ML): Machine learning-based methods mostly require working with a large set of historical data, where input from a data engineer is needed more than a technical expert. With these techniques, it is possible to compute RUL values directly from measurements, but the final result is strongly dependent on the chosen architecture and selected test data. The weakness of the approaches here is the lack of insight into the mechanism of operation of the “black box model”. Methods used in this group include:
  – Artificial neural networks (ANNs)—these are often employed for RUL estimation tasks because of their ability to adapt, handle nonlinearity and accurately approximate target functions and parameters [177]. ANNs mostly omit the process of modeling machine degradation by finding the valid relationship between machine condition and time. This approach is often applied to gearboxes [178], bearings [179] and remaining rotating machinery.
  – Support Vector Machine (SVM)- and Support Vector Regression (SVR)-based methods—these can be applied to the RUL task by using both regression [180,181] and classification [182,183]. They are effective for prediction tasks taking into account the non-linearity of estimated characteristics. They require the use of a suitable representative training set for the created model, but the relatively high computational complexity of the algorithm limits the size of the training set. This group of methods is often used in combination with other techniques to obtain optimal results [184] such as principal component analysis (PCA) for feature reduction [185]; regression and thresholding [186], survival analysis [187,188], hidden markov models (HMMs) [189] or similarity comparison [190].
- Physics of failure: This technique covers computing the remaining useful life of equipment based on a mathematical process or phenomenon model. It requires a high level of expertise to create the model and calculate the degradation characteristics. Calculations treat material properties and stress/load levels to calculate crack growth, deformations, wear, corrosion and other undesirable events [191]. This type of approach usually provides excellent and understandable results. Due to its complexity, it is used in specific cases (i.e., for a particular phenomenon, e.g., boiler wall thickness, progressive corrosion). A popular trend also in the area of predictive maintenance is the development of digital duplicate devices or entire installations called *digital twins*. For example, Aivaliotis et al. [192] presented a methodology of device simulation based on empirical data and calculations of the remaining useful life.

### 4.7. Summary and Comparison of Approaches Used in Prognostic Health Monitoring

The broad spectrum of possible methods used in predictive maintenance tasks makes it difficult to choose the proper one for the particular problem or monitored equipment. Here we try to summarize the described groups of methods for prognostic health monitoring. Table 2 shows use cases and different groups of methods used for prognostic health monitoring. The features and characteristics of the solutions are presented in terms of required resources for implementation and possible use cases. The table uses labels to indicate the strong occurrence of a feature (✓), the absence or contradiction (×) and the occurrence of a feature in certain cases or when it is not dominant (−).

In terms of use cases, the following methods were considered for:

- Fault detection—identifying sensor and device failures, including degraded performance states;
- Anomaly detection or undefined faults at the design stage;
- Fault classification—the capability to diagnose a specific failure mode and to detect multiple defects simultaneously;
- Root cause analysis—the capability to find root causes and analyze failures;
- Remaining useful life prediction—the capability to detect faults in the future over a long-term time horizon.

The resource requirements of the methods considered include:

- Expert knowledge—involvement of domain experts, models, documentation related to the specifics of the process;
- Data science—required knowledge of data processing, statistics and model design, e.g., deep learning;
- Large dataset—the need of having large sets of historical data to train the model;
- Transparency—clarity of model operation and analysis of results.

The choice of the method depends on the specificity of the process. The advantages and disadvantages of the individual methods, together with examples of their applications, are presented in Table 3. Physical models offer the most accurate results, while fuzzy systems, particle and Kalman filters and stochastic methods deal well with noise. Methods based on ANNs and Bayesian networks are used in the case of non-linear data dependencies. Methods that rely on residuum analysis from the analytic redundancy group and ANNs have the best inference for detecting previously undefined faults.

Expert knowledge is especially needed in the case of qualitative methods based on expert systems or physics of failure and in the case of methods that require the design of relations or hierarchization of device states, e.g., fault trees, Markov models, Bayesian networks.

Both a large dataset and data science capabilities are required when using ANNs and stochastic methods (unless there is a representative sample of data).

### 4.8. Prescription

Prescriptive maintenance exhibits the highest degree of analytic maturity of the organization and thus the complexity of the systems. The provided results of prescriptive maintenance are recommendations (prescripts) that guide decisions to the best path using advanced computation and simulation. Prescriptive maintenance encompasses a range of tools and methods that integrate available data and information to optimize a process by providing recommendations or automating the decision process entirely. Data acquisition and integration, optimization and user interaction are critical issues and challenges in this group of maintenance methods.

#### 4.8.1. Data Acquisition and Integration

Modern technologies from the areas of Big Data, Industrial Internet of Things (IIoT) or cloud/fog computing extend the current capabilities of the systems in place. Data can be collected in dedicated repositories to analyze and integrate both production data from technological systems, such as SCADA or DCS and the data from enterprise asset-management or -maintenance systems. Integration and development of systems can occur in the following directions [193]:

- Horizontally—expanding the scope of current areas, analyzing more extensive amounts of data, including Big Data and sharing knowledge in the organization. In particular, it focuses on optimizing the entire supply chain, taking into account customer and supplier data.
- Vertically—combining data from different internal segments to gain knowledge, e.g., machine-acquired data from sensors and technological systems with human-made data from company relational systems.

**Table 3:** Advantages and disadvantages of specific prognostic and health-management techniques.

	Pros.	Cons.	Application
Bayesian Network	easy to understand and transparent encodes expert knowledge used for both RUL and RCA purposes handles uncertainty	complex preparation process both expert and analytical knowledge required finds only known/defined cases	fault detection [158] diagnosis [194,195] scheduling [196] RUL [197,198]
SVM	good modeling of non-linear and linear relationships used for both regression and classification does not require a large learning set	lack of transparency data scientist’s knowledge needed with large datasets, long computation times	fault detection [137,138,185] condition monitoring [199,200]
PCA	handles multidimensional datasets works well with other techniques generalizes the data	loss of some information features lose linking to specific components	fault detection [131,132,201,202]
Expert system	transparent and easy to understand good interaction with domain knowledge no need for a physical process model	advanced models require a strong effort works only with defined cases	fault detection, planning [203] fault detection [204,205,206]
Fuzzy logic	extends the capabilities of the expert system to time series analysis deals with input noise and uncertainty	requires knowledge to apply fuzzy rules	fault detection [122] diagnostics [207]
Physical models	provide precise results for a specific well-known case/process algorithms understandable for industry experts	require considerable modeling effort and extensive domain knowledge	RUL [208] condition monitoring [209]
ANNs	provide the capability to model complex, non-linear relationships no domain knowledge required can be used in conjunction with other techniques provide direct result output	“black box” results may be non-transparent prone to overfitting difficult in determining the uncertainty of results require a large training set	RUL [178,179]
ARIMA	computationally efficient does not require large datasets requires no expert knowledge	short term forecast only sensitive to noise and process variations	RUL [210,211,212,213,214]
HMMs	allow modeling of both time series and stationary data handle incomplete datasets	computationally complex do not detect previously undefined events	fault detection [126,127] RUL [215,216]

#### 4.8.2. Simulation and Optimization

The optimization task in the industry often goes beyond the trivial task of solving a linear objective function. The analyzed problems and physical behavior are often expressed by nonlinear characteristics with a high degree of uncertainty.

An essential issue while designing a solution for optimization tasks is to define the optimization objective correctly. In the industry, the objective may be to increase productivity, energy efficiency, increase the remaining useful life or improve scheduling. It is not always possible to strike a balance between the various objectives, so it is important to understand and define the goal according to the priorities.

The computational complexity and the requirement for online access often require heuristic algorithms to achieve an acceptable solution in a given time. Examples of methods used here, in between deterministic mathematical modeling, include the fields of Artificial Intelligence or evolutionary algorithms:

- Genetic algorithms (GAs): these rely on the mechanism of natural selection, where the genes of the strongest individuals (chromosomes) are passed on to the next generation, allowing the species to survive. The optimization process involves creating a population of individuals and encoding the variables as genes. Each individual represents some way of solving the problem, as evaluated by the fittest function. Appropriate genes are selected in subsequent iterations simulating crossover to bring the solution closer to the optimal value. This optimization method has a wide range of applications. It is very often applied to feature selection and optimization tasks as one of the steps in the data-mining process. Toma et al. [217] presented an example of using GA in feature selection in their algorithm for detecting bearing failure in an engine. More complex applications can be found in the area of distribution grids, with solutions for demand management in smart grids [218] and power flow optimization [219].
- Particle swarm optimization (PSO) algorithms: these are based on simulating the behavior of a flock of birds or a school of fish. Each individual (bird or fish) represents a solution. Unlike the genetic algorithm, traits are not crossed but evolve by following the best candidate. The algorithm is applied in complex optimization and prediction tasks and is often used in the area of renewable energy sources. Jordehi [220] engages the algorithm for the parameter estimation of photovoltaic modules, while in the article [221] concerning wind farms, PSO is used for load flow prediction.
- Ant colony methods (another group inspired by nature): these mimic the behavior of ants that can find the shortest path between the anthill and food by communicating with each other via chemical substances. The algorithm is particularly applicable to scheduling optimization [222,223].
- Dynamic programming (DP): this involves dividing the problem into smaller parts and looking for the overall solution by assembling the results for sub-problems. The approaches used include top-bottom, most often using recursion and bottom-up, solving sub-problems and aggregating, e.g., in an *n*-dimensional table. This group of methods is often used in computationally complex models with many uncertain variables (e.g., variable external factors such as weather) for optimizing the operation and maintenance of energy sources [224,225].
- Reinforcement learning: this is one of the main trends in machine learning alongside supervised and unsupervised methods. Unlike supervised methods, modeling does not involve batch computation of the relationship between output and input. Learning involves continuous interaction of the model (agent) with the dynamic environment in real time and optimizing the process using a defined reward function. Recent articles mention the advantage of real-time process optimization for the chemical industry [226] and water-distribution system [227].

#### 4.8.3. Digital Twin

**Figure 15:**
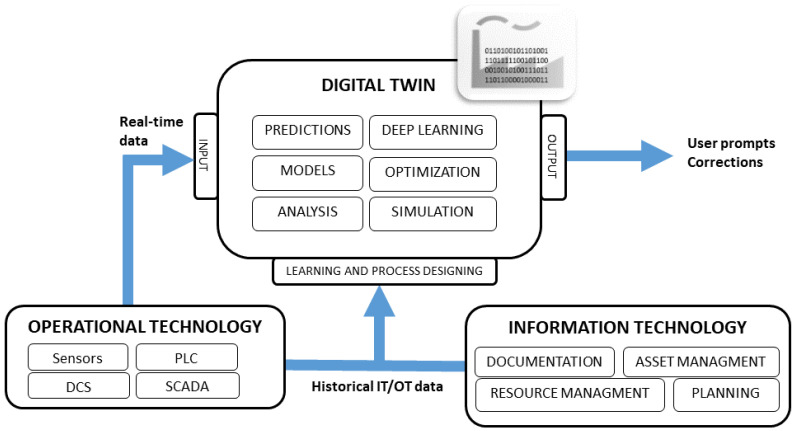
Sample diagram of digital twin.

The digital twin concept often appears in works related to Industry 4.0. The digital twin is an advanced digital model reflecting a particular physical machine, system or plant and enables one to perform advanced calculations and simulations, often combining data-driven and model-based techniques. Initially, the concept was used in aeronautics and aircraft field [228,229], but with time it was adapted to other industries. According to Grieves’ definition [230], a digital twin contains three components: physical components in the real world, virtual models in a virtual space and connections between these virtual and real entities. In practice, this means that digital twins can operate on real-time data, e.g., from sensors or control systems and connect them with historical data to be analyzed in virtual models. The virtual model gives feedback based on simulations and optimizations to the physical device (in the form of recommendations or control instructions). The architecture of a digital twin typical for predictive maintenance and production optimization tasks is shown in Figure 15.

Digital twin through simulation-based optimization is applied in many areas. Power industry applications include areas related to:

- Fault detection and predictive maintenance—in the maintenance area, we can use digital twin for health prediction and remaining useful life [192,231].
- Performance optimization—this supports technical and economic modeling of coal-fired power plant units and investigates cost-effective solutions to improve their thermal efficiency and operational performance [232].
- Education and training—modern solutions enable one to perform training in simulated conditions, allowing one to imitate situations of disasters and breakdowns while maintaining safety for employees [233].
- Energy consumption optimization—digital twin implements reorganization of energy consumption patterns to avoid peak demand while reducing energy costs [234].

## 5. Other Technology Enablers

Apart from the approaches mentioned in the previous section, other technological approaches, including Industrial Internet of Things, Big Data, Cloud and Edge computing, augmented reality, radio-frequency identification and 3D printing, accelerate progress in the maintenance domain. This section briefly explains them.

### 5.1. Industry 4.0 Concepts

Industry 4.0 is a term associated with applying modern Information and communications technologies (ICTs) in industry, unlocking new opportunities focused on device interoperability, Artificial Intelligence (AI) and digitization [235,236]. The primary drivers of the process are the reduction of costs for sensors, communication devices, data storage and the continued development of technologies based on the Internet of Things, Big Data and Artificial Intelligence.

**Figure 16:**
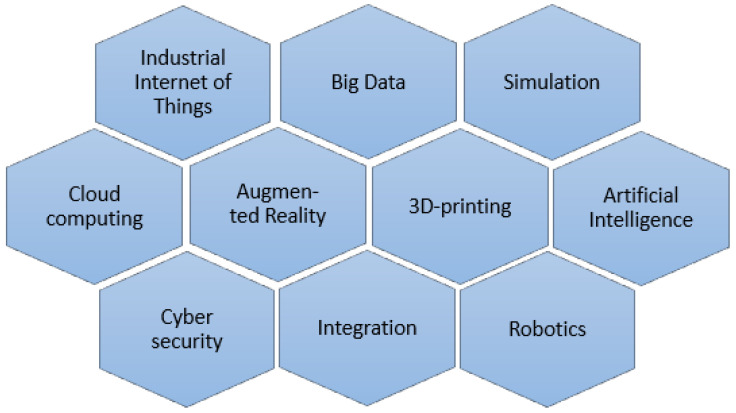
Main pillars of Industry 4.0.

Concepts strongly related to the development of Industry 4.0 are as in Figure 16:

- Big Data [237]: this means large volumes of structured, semi-structured and unstructured data that require specialized technologies to enable efficient storage, fast processing and analysis, to obtain higher business value;
- Augmented reality [238]: this refers to technology that provides access to information, communication and visualization through dedicated glasses;
- Autonomous robots [239]: this relates to using robotics and Artificial Intelligence to create machines/devices that communicate with other robots or humans while performing specific tasks;
- Three-dimensional printing [240]: this provides the ability to create physical objects, parts and prototypes from a virtual design;
- Simulation [241]: this provides the ability to predict future conditions and outcomes for various scenarios within the equipment, installations and even the entire power plant;
- Systems integration [242]: this means linking data from multiple plant-level systems and external sources;
- Cloud computing [243]: this relates to scalable and on-demand data processing and analysis using a network of interconnected servers that give a satisfactory performance;
- (Industrial) Internet of Things [244]: this refers to technologies that provide communication and data exchange between machines and people in an industrial environment;
- Cyber-security [245]: this points to technologies protecting the system against unauthorized access to data and taking control over the device.

These components enhance operability through the capability to remotely control, determine equipment health, predict failures, proactively maintain and deploy real-time applications, leading the power plant toward a “smart” factory. A summary of technologies related to Industry 4.0 is presented in Table 4.

### 5.2. Industrial Internet of Things

Rapidly evolving communication technologies enable easier access, integration and industrial data analysis. This evolution has a particular impact on predictive maintenance because it allows the data from sensors or actuators to be used for many more tasks than initially intended. The Industrial Internet of Things provides a bridge between a physical factory (consisting of sensors, measurements and events) and a virtual collection of models, machine learning and Artificial Intelligence. The combination of data fusion and communication enables applications such as the integration and collaboration of Autonomous Guided Vehicles with the production environment [265,266].

Communication protocols such as Message Queuing Telemetry Transport (MQTT), Constrained Application Protocol (CoAP) or OPC Unified Architecture (OPC UA) ensure secure connections in industrial environments and various types of communication such as machine to machine (M2M), client-server. The aspect of ensuring security in IoT solutions is particularly important in the energy area. Many works are devoted to applying solutions to ensure cyber security by creating dedicated architectures of IoT systems and using deep learning techniques for detecting threats [267]. An example of notable solution use RF (radio-frequency) fingerprinting techniques to identify and legitimize devices on the network [268].

### 5.3. Big Data

Big Data is generally defined as data in large volumes, requiring special technologies to handle them. Collecting, storing and analyzing data in this form exceeds the capabilities of traditional technologies such as relational databases. Big data properties are most often described in terms of multiple “Vs”:

- Volume: this refers to the massive amount of data. In the industrial environment, it concerns machine-generated data from devices, sensors or security systems. The enhanced capabilities offered by the IoT make it even more important to develop technologies that can manage large volumes of data.
- Velocity: this means fast data generation. It is most often associated with the ability to process streaming data in real time.
- Variety: this refers to data types that can be processed. It does not limit itself to work only on structured tabular datasets but enables processing either structured (numbers, dates, strings), semi-structured (graphs, trees, XMLs) or unstructured (logs, videos, images) datasets.
- Value: this refers to the value of the information potential that results from applying algorithms and analysis on datasets.
- Veracity: this determines the reliability of the information by analyzing its source and associated metadata. An example of an indicator of the relevance of information could be the last update time.

Such data appear in many areas of industry and maintenance. With the help of dedicated data repositories or cloud platforms, we can collect, process, transform and query Big Data. Modern systems not only allow for data warehousing [269] or data stream analysis [270] but enable assessment of data quality and approximate information searching in the absence of parts of the dataset [271,272].

### 5.4. Cloud/Edge Computing

Current industrial systems adopt new architectures to provide high scalability and computing power, low latency or security as needed [273]. A wide range of applications allows for both data stream analysis and batch processing of large historical data. However, we can perform these operations in various computing models, including:

- Edge computing [274]—this allows for data processing and computations close to the device (for example, performing the Wavelet transform, the Fast Fourier transform or data merging and aggregation [266]). This approach handles data velocity but has limited storage, passing collected and computed data for further processing.
- Fog computing [275]—this moves data processing from the device itself to fog nodes in the network. The approach also provides lower latency computing on a slightly larger scale.
- Cloud computing [276]—this employs cloud technologies and IoT hubs (for example, Azure IoT, AWS IoT), providing a highly scalable environment and computing power. It includes services for end-users that perform analytical and monitoring tasks, making security issues and latency a bit more complicated.

Due to the commercial nature of the cloud services provided, an important issue is the reasonable use of resources in pricing schemes. A noteworthy solution uses heuristic-based polynomial time policies to optimize resources to be reserved [277]. A brief comparison of the edge and cloud approaches is shown in Table 5.

### 5.5. Augmented Reality

Augmented reality (AR) is a technology that allows combining real images with virtual objects, giving the recipient a larger amount of information and introducing the possibility of interaction. AR systems provide promising results in various maintenance areas, such as training, inspection or repairing processes. Examples of applications may include:

- Training. This concerns applications of virtual and augmented reality in crew training. A trained employee moves in the designed 3D model of the environment performing subsequent procedures in accordance with the training tasks. It is beneficial in industries with a serious risk of exposure to life-threatening situations.
- Data access. AR technology can provide an interface to existing systems such as document management, Computerised Maintenance Management System (CMMS) or work order management. Many practical applications relate to the presentation of instructions and device documentation in electronic form. This speeds up operation time by avoiding printed documents and searching for information, especially in complex installations such as a power plant.
- Inspection support. Through integration with process data, processing systems view sensor data and device status online. Mobility also allows collecting information in a different way than writing it down in a notebook—by taking pictures or recording sound. Many solutions are based on online collaboration between the technician and the remote expert. In the case of complex repair work, the expert can see the same as the technician can see on-site, give instructions and display documentation and other materials.

### 5.6. Radio-Frequency Identification (RFID)

Radio-frequency identification (RFID) has long been used in asset-maintenance and -management tasks in many industries. The technology is based on tagging objects with RFID tags and wireless reading of the information stored in the tags. The idea of the operation is similar to barcodes, but the undoubted advantage of RFID is the ability to read data from a long distance and collectively. For preventive inspections, work can be done faster and with more ergonomics when automated data collection replaces manual sheet filling [256]. The unambiguous identification of devices allows for better data quality and improves inventory processes.

### 5.7. 3D Printing

One of the emerging technologies associated with Industry 4.0 is 3D printing (also called additive manufacturing). The technology allows building a physical object or part using a 3D printer and a virtual design created in CAD (Computer-Aided Design) software. The technology can find promising applications for designing and prototyping in modern manufacturing, also in the healthcare and medical industry [278,279,280]. In the field of maintenance, we can use new possibilities to recreate damaged parts [259,260]. The ability to manufacture any component reduces inventory costs and procurement time, giving new perspectives to support cost-effective maintenance strategies [261,262]. A summary of technologies related to Industry 4.0 is presented in Table 4.

## 6. Summary and Discussion

This section summarizes the methods and applications described and compares them with the specifics of the work and the requirements of the energy industry. Finally, the challenges and realities section identifies areas for potential research to enable better application of the results in practice.

### 6.1. State of the Art

The literature review confirms that many solutions, both in academic papers and real-world applications, can support the maintenance process. Despite its considerable development potential, the energy industry cautiously benefits from new technologies, applying new solutions in a narrow range. Corrective and preventive strategies are still the main approaches used in the energy sector. Complex solutions, including advanced technologies such as Big Data, predictive maintenance, are usually offered as an integral part of the system or an additional service by the control system provider. In the area of predictive maintenance, examples include the Predix™platform [281] offered by General Electric or the ABB Ability™ [282] offered by ABB. In the area of production optimization, VALMET offers a solution to improve processes, for example, combustion, fuel feeding [283]. Why are proven solutions in other industries and methods that achieve good results in laboratories not applicable in the real world?

### 6.2. Energy Industry Specificity

In general, power plants in operation have equipment designed to operate for several years, including control systems, so integration with new solutions is a technological barrier if not considered at the design stage. Changes and implementations considered for investment must present an appropriate business case and the return on investment factor. In the energy sector, high availability has for years been achieved through the appropriate design of production and logistics processes, e.g., duplication of critical equipment functions (switching to a backup device in case of failure) or storage of components such as motors or pumps to quickly replace a defective device. The profits from smart manufacturing appear if the adopted approach will allow limiting traditional preventive actions, which, however, is related to taking a certain risk.

In the case of indirect influence on profits, the share of the implemented solution is also debatable. In the case of performance improvement, it is easy to make a mistake or statistical fallacy; e.g., what influence does the predictive maintenance system, crew training or overhaul have on the bottom line?

### 6.3. Challenges and Realities

Technological progress is opening up new opportunities in the industry to use data analytics to support manufacturing processes, thus complementing the benefits of existing expert systems and data repositories. However, many obstacles prevent analytical solutions from being developed or experiments operating in laboratory environments from being applied in production environments. One of the challenges is to meet the requirements of the end user, taking into account the specific working conditions in the power industry. The solutions should be carefully designed in terms of who will be using them. Systems supporting operational work should consider information noise and the stressful nature of operators’ work. In contrast, solutions that help in planning repair works should be appropriately integrated with enterprise systems visualizing financial implications and possible risks. Effective implementation of the system supporting the work of the operator working in the open loop architecture (i.e., giving prompts in real time) requires paying special attention to elements such as visualization or human–machine interface. Another field for researchers is to take care of the problem of uncertainty in decision support systems. This is of particular importance when decisions are made under stressful conditions and require compliance with applicable procedures.

A significant barrier encountered in the energy industry is the alignment of systems with internal safety and regulatory requirements. Power plants are often part of critical infrastructure, which brings additional requirements in the areas of cyber security, physical security and providing operational continuity. Special attention is being paid to nuclear power plants because of the environmental aspects of possible incidents. This increases the cost of implementation and infrastructure (e.g., may require on-premise instead of cloud computing), requires logical or physical separation of network environments and hardens development options. On the other hand, the requirements to ensure cyber-security and physical security of power plant facilities open up opportunities to develop research and solutions towards the use of Industry 4.0 technologies such as the Internet of Things or Artificial Intelligence to provide better protection against threats.

The power industry is the area where stability and security are prioritized over the implementation of innovations and where continuous improvement of competitive advantage is not required. Innovative solution implementations should engage branch engineers both to align solutions with the industry properly and, most importantly, to have a good understanding of data-driven and Industry 4.0 methods.

Initiation of large-scale change may be difficult, may trigger the implementation of new technologies and may include the following: aging workforce or energy transition needs, meeting environmental requirements.

## 7. Conclusions

Modern technologies that enable the acquisition, integration and analysis of new industrial data sources provide new possibilities for supporting maintenance processes. A particularly wide field for application is predictive maintenance, where classic methods requiring specialized equipment and expert analysis are replaced by Artificial Intelligence inference based on existing metering, widespread digitization allowing online condition monitoring of equipment and feedback both in the form of operator interaction and a fully automated prescriptive system. The proposed classification of methods with a description of properties and required resources gives an overview of potential solutions for each use case. The presented methods allow implementing tasks related to fault detection and identification depending on the potential of possessed expert knowledge, data resources, the quality of data and equipment metering. The main contribution of this part of the article to this issue is to the present state of the art for considering the transformation of existing processes and routines in the area of asset management. The presented applications and use case approaches can be utilized in similar solutions or encourage the initiation of research and development tasks or proof of concept projects.

The combination of inference from process data analysis with corporate data from the areas of finance and asset management allows for optimal selection of the maintenance strategy and supervision of its implementation. The classic approaches presented in the article can help innovators and data scientists find new directions to fill the technology gap and better align their research with the requirements of interested stakeholders.

Other enablers associated with the development of technologies on the verge of Industry 4.0, such as the Internet of Things, RFID, augmented reality, are also successfully applied in the maintenance area, providing additional support during inspections and automating previously manual tasks.

Summarizing, the article is an extensive resource to motivate practitioners to learn sophisticated analysis methods and to point researchers to potential directions for further development.

## Figures and Tables

**Table 2:** Use cases for prognostic health-monitoring methods. The symbols mean: The strong occurrence of a feature (✓), the absence or contradiction (×) and the occurrence of a feature in certain cases or when it is not dominant (−).

	FTA	Expert Systems	Fuzzy Systems	Analytical Redundancy	QTA	Statistic	Stochastic	ANN
Fault detection	✓	✓	✓	✓	✓	✓	✓	✓
Anomaly/new fault detection	×	×	×	✓	×	✓	×	✓
Fault classification	✓	✓	✓	×	✓	−	✓	✓
Root cause analysis	✓	−	−	−	−	−	✓	−
Remaining useful life	×	✓	✓	−	−	✓	✓	✓
Expert knowledge needed	✓	✓	✓	×	×	×	−	×
Data science/statistic knowledge needed	×	×	×	×	−	✓	✓	✓
Large dataset needed	×	×	−	−	−	−	✓	✓
Transparency	✓	✓	✓	−	✓	−	✓	×

**Table 4:** Industry 4.0-related technologies and application areas.

	Enhances	Related Technologies	Refs.
IIoT	Data acquisition New data streams Connectivity Interoperability	IoT Gateway, IoT hub, CoAP, MQTT, XMPP	[246,247]
Big Data	Data storage Stream processing Unstructured data	Hadoop, Spark, Kafka, splunk, NoSQL	[248,249,250]
Cloud Computing	Data analysis Applications Infrastructure	Edge/fog computing, Service models (IaaS, PaaS, SaaS)	[251,252,253]
Augmented reality	Inspections Communication Mobility	Smart glasses, Natural Language Processing (NLP), Geolocalization, Gesture recognition	[254,255]
RFID	Inspection Inventory Data collection	Active/passive tags, Bulk reading	[256,257,258]
3D Printing	Designing Replacements	Computer-aided design, 3D scanning	[259,260,261,262]
Digital Twin	Simulation Virtualization Optimization	Machine learning, simulation software (e.g., ANSYS)	[263,264]

**Table 5:** Comparison of cloud and edge computing. Explanation of symbols: ✓- enough, ✓✓- good, ✓✓✓- very good.

	Cloud Computing	Edge Computing
Access	wireless	wireless
Availability	✓✓✓	✓
Capacity	✓✓✓	✓
Architecture	centralized	distributed
Latency	✓	✓✓✓
Scalability	✓✓✓	✓
Security	✓	✓✓✓
Mobility	✓✓	✓✓✓

## Abbreviations

The following abbreviations are used in this manuscript:

4M	Man, Machine, Methods, Materials
AADL	Architecture Analysis and Design Language
AI	Artificial Intelligence
ANN	Artificial neural network
AR	Augmented Reality
ARIMA	Autoregressive integrated moving average
ARMA	Autoregressive–moving average
ARMAX	AutoRegressive Moving Average with eXogenous input
AWS	Amazon Web Services
CAD	Computer-Aided Design
CBR	case-based reasoning
CMMS	computerized maintenance management system
CNN	Convolutional Neural Network
CoAP	Constrained Application Protocol
CUSUM	Cumulative Sum
CWT	continuous wavelet transform
DC	direct current
DCS	distributed control system
DP	Dynamic programming
DWT	discrete wavelet transform
EAM	enterprise asset management
EMD	empirical mode decomposition
ERP	Enterprise Resource Planning
FDI	fault detection and identification
FMEA	Failure Mode and Effect Analysis
FTA	Fault tree analysis
GA	Genetic algorithm
HHT	Hilbert–Huang transform
HMM	hidden Markov model
IaaS	Infrastructure as a Service
ICT	Information and communications technologies
IIoT	Industrial Internet of Things
IMF	intrinsic mode function
KPI	Key Performance Indicator
LSTM	long short-term memory
MCSA	Motor Current Signature Analysis
MES	Manufacturing Execution System
ML	Machine learning
MLP	multi-layer perception
MQTT	Message Queuing Telemetry Transport
MTBF	mean time between failure
MTTF	mean time to failure
NLP	Natural Language Processing
OPC UA	OPC Unified Architecture
OT	Operational Technology
PaaS	Platform as a Service
PCA	principal component analysis
PdM	Predictive maintenance
PHM	Proportional hazard model
PLC	programmable logical controller
PLS	partial least squares
PSO	Particle swarm optimization
PV	photovoltaic
QTA	Qualitative trend analysis
RBF	radial basis function
RCA	Root cause analysis
RCM	Reliability-Centered Maintenance
RF	Radio frequency
RFID	Radio-frequency identification
RNN	recurrent neural network
RPN	risk priority number
RUL	remaining useful life
SaaS	Software as a Service
SCADA	Supervisory Control And Data Acquisition
STFT	Short-time Fourier Transform
SVM	support vector machine
SVR	Support Vector Regression
TPM	Total Productive Maintenance
UML	Unified Modeling Language
WT	Wavelet transform
WVD	Wigner–Ville Distribution
XML	Extensible Markup Language
XMPP	Extensible Messaging and Presence Protocol
